# Influence of age and gender in the sensory nerve fibers excitability

**DOI:** 10.1002/brb3.2467

**Published:** 2021-12-28

**Authors:** André Caetano, Pedro Pereira, Mamede de Carvalho

**Affiliations:** ^1^ Faculdade de Medicina Instituto de Fisiologia Instituto de Medicina Molecular Universidade de Lisboa Lisbon Portugal; ^2^ Department of Neurology Centro Hospitalar de Lisboa Ocidental, Hospital de Egas Moniz Lisbon Portugal; ^3^ Department of Neurology Hospital Garcia de Orta Almada Portugal; ^4^ Department of Neurosciences Centro Hospitalar de Lisboa Norte, Hospital de Santa Maria Lisbon Portugal

**Keywords:** axonal excitability, reference values, sensory nerve, threshold tracking

## Abstract

**Objectives:**

To assess the influence of age and gender on sensory nerve axonal excitability parameters.

**Methods:**

Thirty‐three healthy subjects (21 women) were included, with a mean age of 34.6 (range 21–76). Median sensory nerve excitability measurements (index finger) were performed using the TRONDNF nerve excitability protocol of the QTRAC program.

**Results:**

Peak sensory nerve action potential (SNAP) amplitude was significantly higher among women (27.1 vs. 9.2 μV; *p* = .022), and strength–duration time constant (SDTC) was significantly higher in men (0.7 vs. 0.5; *p* = .011), not dependent on age. Greater age was negatively correlated with resting *I*/*V* slope, not dependent on gender (*r* = –0.4; *p* = .024). No other changes in excitability properties with increasing age were found.

**Conclusions:**

Physiological features like as age and gender do not have a relevant impact on sensory nerve excitability measurements, which can have implications regarding pharmacological treatments.

## Graphical abstract

To investigate the influence of age and gender on sensory nerve axonal excitability parameters.

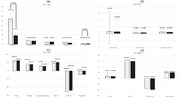

## 1 INTRODUCTION

The assessment of axonal excitability using threshold tracking methods (Bostock et al., 1998; Kiernan et al., 2000a,b) was initially developed for the evaluation of motor nerves and subsequently adapted for sensory nerve evaluation (Kiernan et al., 2001). There have been several reports on the effect of age and gender on motor nerve excitability parameters (Bae et al., 2008; Casanova et al., 2014; Jankelowitz et al., 2007; Lai et al., 2015; McHugh et al., 2011), namely a reduction in motor amplitude, accommodation half‐time, stimulus–response slope (Casanova et al., 2014), and in superexcitability (Bae et al., 2008; Jankelowitz et al., 2007; McHugh et al., 2011), as well as a flattening of the normalized stimulus response curve and reduction in threshold change following strong hyperpolarizing currents (McHugh et al., 2011).

With regards to sensory nerve excitability changes with age, previous studies on mice sensory nerves have shown that aged mice had an increase in stimulus required for 50% of maximal amplitude of the sensory nerve action potential as well as rheobase, as well as a significantly lower early hyperpolarizing threshold electrotonus (TEh), namely TEh (20–40 ms) and TEh (peak), as well as superexcitability, while late subexcitability decreased nonsignificantly (Banzrai et al., 2016).

So far few studies have been performed on age‐dependent changes human sensory nerves, with conflicting results, as some reports have shown changes which may suggest less inactivation of transient sodium channels (Lai et al., 2015), whereas other reports suggest that age alone does not seem to be a significant factor in excitability changes of sensory nerves (Kiernan et al., 2001).

We aimed to further improve knowledge on the influence of both age and gender on human median sensory nerve excitability parameters of healthy subjects.

## 2 MATERIALS AND METHODS

### 2.1 Subject selection

We recruited healthy subjects of different ages and both genders. Only subjects without clinical or electrophysiological evidence of peripheral nerve disorders, without symptoms suggestive of carpal tunnel syndrome, and not taking drugs that could affect nerve excitability were selected for the study. We ensured that the median sensory nerve action potential was normal (using antidromic conventional nerve conduction studies). Threshold tracking studies were performed on the right arm of every subject.

### 2.2 Peripheral nerve excitability assessment—threshold tracking

Excitability studies were performed according to previously published standard protocols (Bostock et al., 1998; Caetano et al., 2019; Kiernan et al., 2001, 2000b). Every subject was seated in a relaxed position, with the right arm resting on a pillow, and when needed, investigated hand was warmed either by a heater device or a hot water bag placed underneath the right arm (Caetano et al., 2019). The temperature was kept at a minimum of 31°C during the procedure, with regular checks after each step of the protocol. Whenever the temperature dropped below the established minimum, the test was interrupted and the hand warmed again (Caetano et al., 2019). Excitability measurements were performed using the TRONDNF nerve excitability protocol of the QTRAC program (Professor Hugh Bostock, Institute of Neurology, Queen Square, London, UK). The EMG signal was recorded through a D440‐2‐Two Channel Isolated Amplifier (Digitimer, Welwyn Garden City, UK) connected to a NeuroLog System (Digitimer, Welwyn Garden City, UK) and filtered between 2 Hz and 10 kHz. The active electrode was placed overlying the proximal phalanx of the index finger and the reference on the distal phalanx of the second finger (20 mm diameter disk, E. K50430‐001, Digitimer, Welwyn Garden City, UK). Stimulus waveforms were generated by the test computer and converted to current by a DS‐5 isolated linear bipolar constant‐current source (Digitimer, Welwyn Garden City, UK) with a maximal output ±50 mA. The stimulus currents were applied via nonpolarizable electrodes (20 mm diameter disk, E. K50430‐001, Digitimer, Welwyn Garden City, UK) with the active electrode over the nerve at the wrist and the reference electrode ∼10 cm proximal at the lateral region of the forearm. The amplitude of the sensory nerve action potential (SNAP) was measured from peak‐to‐peak. For all tracking studies, the target SNAP was set to 40% of the peak response (Kiernan et al., 2000a,b). The overall excitability variables used are described below.

### 2.3 Variables

Using previously published protocols (Bostock et al., 1998; Kiernan et al., 2000a,b), we obtained the following excitability variables:

- Strength–duration time constant (SDTC), inferred from the relationship between threshold current and stimulus duration, and Rheobase, the threshold for a current of infinitely long duration. Both (SDTC and rheobase) are calculated by measuring threshold for stimuli from 0.2 to 1 ms and plotting stimulus charge versus duration.
- Threshold electrotonus (TE), which measures the threshold changes produced by subthreshold depolarizing or hyperpolarizing currents of 100 ms duration and 20% and 40% (depolarizing [TEd]) and −20% and −40% (hyperpolarizing [TEh]) of the control threshold current; subsequently, the threshold is tested at different time points during and after the polarizing currents.
- Recovery cycle, which is investigated by a double stimulation technique where a supramaximal conditioning stimulus is followed by a submaximal test stimulus, with a variable interstimulus interval (2–200 ms), to evaluate the refractory, supernormal and late subnormal periods.
- Current–threshold relationship (*I*/*V*), which describes the maximal extent of threshold changes from 200 ms polarizing currents, with a strength from +50% to –100% of the resting threshold current.

Using these variables, several excitability parameters were determined and used for statistical analysis (listed in Table 1).

**TABLE 1:** Excitability parameters according to gender

	Female (*n* = 21)	Male (*n* = 12)	*p*
Age, mean (range)	34.6 (21–76)	41.5 (21–68)	ns**
Excitability parameters (mean ± SE)			
Peak SNAP (μV)	27.1 ± 6.4	9.2 ± 3.7	**.022** *
Stimulus for 50% SNAP (mA)	4.1 ± 0.8	3.6 ± 0.3	ns*
Stimulus response/slope	2.3 ± 0.1	2.6 ± 0.2	ns**
Rheobase (mA)	1.9 ± 0.4	1.4 ± 0.1	ns*
Strength duration time constant	0.5 ± 0.02	0.7 ± 0.1	**.011** *
*I*/*V* parameters (mean ± SE)			
Resting *I*/*V* slope	0.6 ± 0.03	0.5 ± 0.04	ns*
Minimum *I*/*V* slope	0.2 ± 0.01	0.2 ± 0.01	ns**
Hiperpolarizing *I*/*V* slope	0.4 ± 0.05	0.4 ± 0.06	ns*
Threshold electrotonus parameters (mean ± SE)			
TEd (peak)	61.6 ± 0.9	62.5 ± 3.6	ns*
TEd20 (peak)	42.4 ± 1.5	28.1 ± 4.1	ns**
Accommodation half time (ms)	29.7 ± 1.6	28.1 ± 4.1	ns*
TEd (90–100 ms)	47.6 ± 0.9	52.3 ± 4.5	ns**
TEh (90–100 ms)	−127.43 ± 5.1	−120.4 ± 5.8	ns**
TEd (undershoot)	−22.2 ± 1.1	−22.7 ± 1.3	ns**
Recovery cycle parameters (mean ± SE)			
RRP (ms)	3.6 ± 0.2	3.6 ± 0.2	ns**
Refractoriness at 2.5 ms (%)	32.1 ± 5.7	29.7 ± 5.3	ns**
Superexcitability (%)	−17.7 ± 1.7	−19.4 ± 1.7	ns**
Subexcitability (%)	9.9 ± 0.5	10.2 ± 0.9	ns**

*I*/*V*: current–threshold relationship; ns: nonsignificant; RRP: relative refractory period; SE: standard error; SNAP: sensory nerve action potential; TE: threshold electrotonus.

* Independent samples *t*‐test (normal distribution).

** Mann–Whitney *U* test for independent samples (nonnormal distribution).

### 2.4 Statistical analysis

We tested for normal distribution of the excitability parameters using the Shapiro–Wilk test. Comparison of parameters between genders was performed using independent samples *t*‐test and Mann–Whitney *U* test according to normality (*α* = .05). We tested for correlation between age and excitability parameters using Pearson and Spearman correlation, respectively, for normal and nonnormal distributions. To assess age‐related changes of excitability parameters, we applied a multilinear regression model using age and gender as independent variables. Significance was set at *α* = .05.

The analysis was performed using IBM SPSS version 24.0 (IBM, Armonk, NY, USA). The study protocol was approved by the Ethics Committee of the Centro Académico de Medicina de Lisboa.

The data that support the findings of this study are available from the corresponding author upon reasonable request.

## 3 RESULTS

**FIGURE 1:**
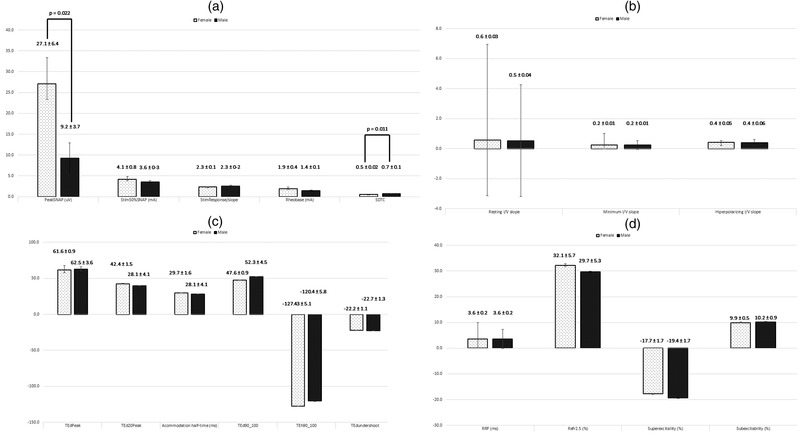
Results

We recruited 33 healthy subjects, among which 21 (63.6%) were female, with mean age of 34.6 (range 21–76), and 12 (36.4%) were male, with a mean age of 41.5 (range 21–68). There were no significant differences between genders regarding age (*p* = .219). In terms of excitability parameters, peak SNAP amplitude was significantly higher among female subjects (27.1 vs. 9.2 μV; *p* = .022), and SDTC was significantly higher among male subjects (0.7 vs. 0.5; *p* = .011). The remaining evaluated parameters did not show any differences between genders (Figure 1). There was a statistically significant negative correlation between age and resting *I*/*V* slope (*r* = −0.4; *p* = .024). In the multilinear regression model, SDTC was independently associated with gender (*p* = .005), with a slope coefficient of 0.2 (95% CI: 0.074–0.392) (Figure 1). However, no other significant changes was confirmed.

## 4 DISCUSSION

Concerning gender‐related differences, and as previously reported (Bolton & Carter, 1980; Kiernan et al., 2001), peak SNAP amplitude was higher among female subjects. This is probably the result of female subjects having digits of smaller circumference, and thus digital nerves being closer to the recording ring electrode (Bolton & Carter, 1980). On the other hand, SDTC was higher among male subjects, which had been previously reported on motor nerve excitability measurements (Bae et al., 2008; Yerdelen et al., 2006). Of the remaining measurements, no other gender‐related differences were found.

Regarding age‐related changes, we found that higher age was negatively correlated with resting *I*/*V* slope, indicating a lower resting conductance in older subjects. The multilinear regression model did not confirm an independent association between age and excitability parameters, suggesting that there is an interaction between gender and age with resting *I*/*V* slope.

Lai et al. (2015) found that in sensory nerves, excitability there was a decreased peak response, increased threshold to 50% of maximal response, increased rheobase, increased threshold during early and late hyperpolarization (TEh [20−40 ms] and TEh [90−100 ms]), decreased relative refractory period, and decreased refractoriness, in the elderly. Although it was proposed that this could represent less inactivation of transient sodium channel, the small *R* values argued against a very potent correlation. On the other hand, Kiernan et al. (2001) had previously reported that age‐related changes in sensory nerve excitability were very limited and restricted to threshold for the 0.5 ms stimulus, rheobase, and stimulus–response slope, suggesting that a modification in tissue impedance could be the reason for these changes rather than axonal membrane excitability alteration. In fact, they did not disclose any significant age‐related effect on most of the excitability parameters (Kiernan et al., 2001).

Regarding our results, lower SDTC in females could be related to anatomical variances (smaller finger diameter) or different tissue properties (Kiernan et al., 2001), but hormonal influence cannot be fully disregarded (Möller & Netzer, 2006). Our results indicate that resting I/V slope of the sensory fibers can be influenced by age. The very minor effect of age and gender on sensory excitability measurements indicate that these physiological features are not critical concerning pharmacological interventions on axonal channels for treating neuropathic pain.

Our study has a number of limitations, in particular, the number of subjects, and only one sensory nerve was investigated. However, there are quite a few studies investigating this subject. Contrary to the stronger evidence regarding age‐related changes in motor nerve excitability properties, our study did not find significant age‐related changes in sensory nerve axonal excitability.

## CONFLICT OF INTEREST

The authors report no conflicts of interest.

## AUTHOR CONTRIBUTIONS

AC, PP, and MdC conceived the original idea for this study. The study design was planned by AC, PP, and MdC. AC and MdC prepared the manuscript with repeated revisions commented on and amended by PP. All authors were involved in the interpretation of the results.

## FUNDING INFORMATION

This study was not supported by any grant.

### PEER REVIEW

The peer review history for this article is available at https://publons.com/publon/10.1002/brb3.2467
